# Supplementary material for: Epstein–Barr virus particles induce centrosome amplification and chromosomal instability
Source: Nat Commun. 2017 Feb 10;8:14257. doi: 10.1038/ncomms14257 (PMC5309802; doi:10.1038/ncomms14257)
Supplement: Supplementary Information — Supplementary Figures [file ncomms14257-s1.pdf]

Supplementary figures

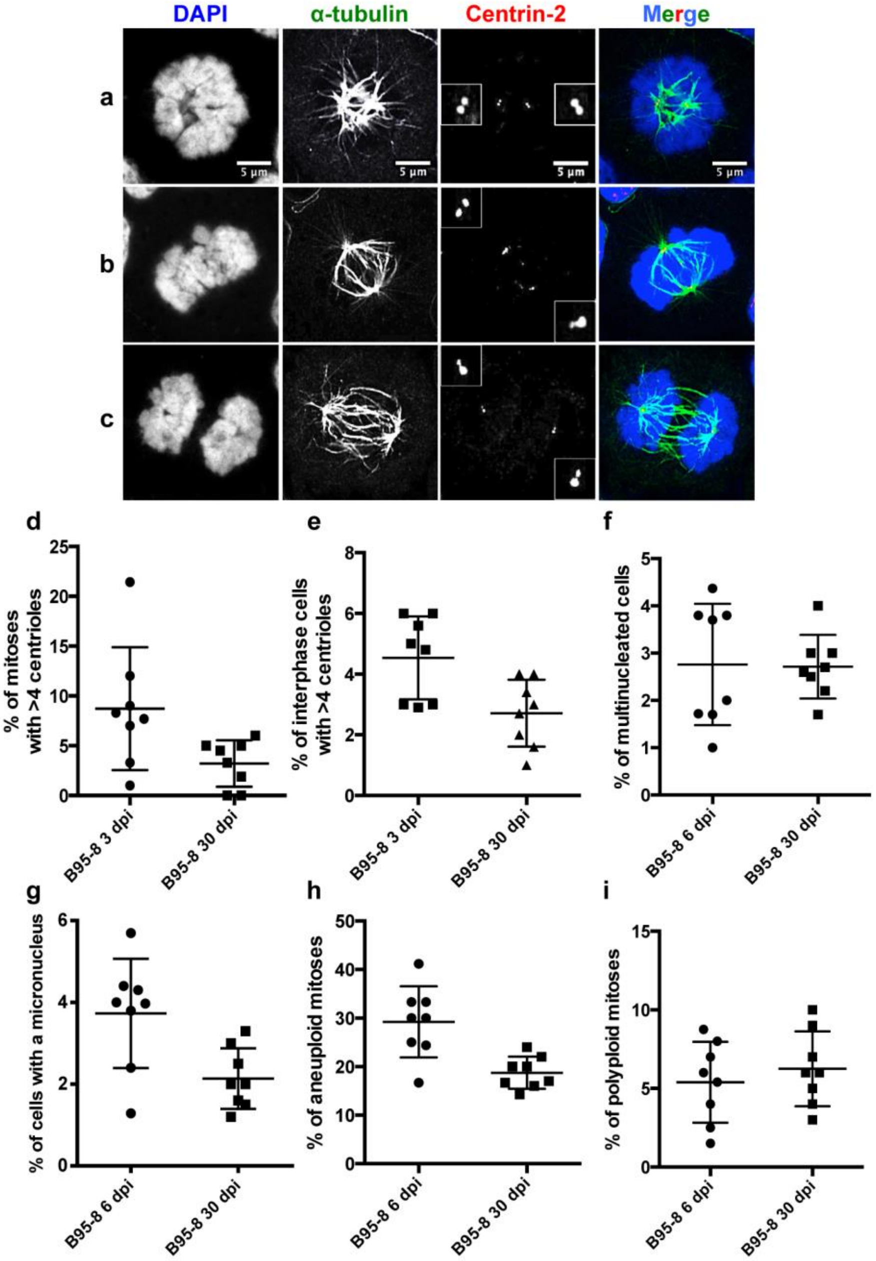

Supplementary Figure 1. B cells stimulated with pokeweed mitogen display normal mitotic figures but not cells infected with B95-8. The figures show cells stimulated with pokeweed mitogen. Cells in prometaphase (a), metaphase (b) and anaphase (c) were stained with antibodies against alpha-tubulin and centrin-2 to visualize the mitotic spindle and the centrioles. Scale bar, 5 µm. (d to i) Rate of chromosomal instability in cells transformed by B95-8 EBV at day 3, 6 or 30 post-infection. This figure summarizes the frequency of bipolar mitoses organized around more than 4 centrioles (d) of interphase cells with more than 4 centrioles (e), of multinucleated cells (f), of cells carrying one or several micronuclei (g), of aneuploid mitoses (h), of polyploid mitoses (i). We have analysed 8 samples. For each sample, at least 100 mitoses and 500 interphase cells were analysed. Error bars represent the mean with s.d. dpi: days post-infection.

a

Sample	Virus	Aneuploidy rate	Clonality	Deletion rate	Translocation rate
1	M81WT	5/20	no	1/20	0/20
2	M81WT	5/20	no	0/20	0/20
3	M81WT	9/20	no	0/20	0/20
	Average	31.7%	0/3	1/3	0/3
4	M81/ΔZR	5/20	no	0/20	0/20
5	M81/ΔZR	5/20	no	1/20	3/20
6	M81/ΔZR	6/20	no	0/20	0/20
	Average	26,7%	0/3	1/3	1/3

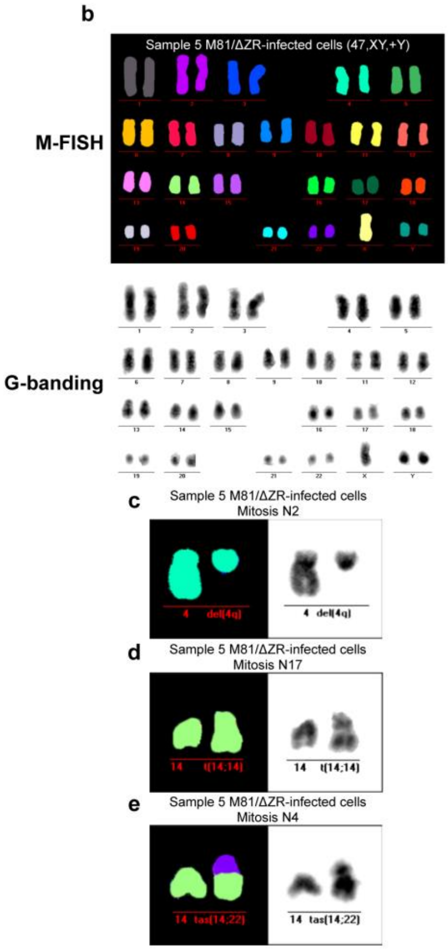

f

Sample 1 M81-infected cells

Mitosis	Karyotype
1	46,XY
2	45,XY,-17
3	46,XY
4	46,XY
5	46,XY
6	46,XY
7	46,XY
8	46,XY
9	46,XY
10	42,XY,-10,-18,-22,-22
11	46,XY
12	46,XY
13	46,XY
14	46,XY
15	45,XY,-17
16	45,del(1q),-10
17	46,XY
18	46,XY
19	45,XY,-19
20	46,XY

Sample 2 M81-infected cells

Mitosis	Karyotype
1	46,XY
2	43,XY,-6,-7,-17
3	46,XY
4	46,XY
5	46,XY
6	46,XY
7	46,XY
8	40,XY,-7,-11,-12,-16,-17,-18
9	43,XY,-17,-19,-21
10	45,XY,-21
11	46,XY
12	46,XY
13	46,XY
14	46,XY
15	46,XY
16	46,XY
17	46,XY
18	46,XY
19	46,XY
20	43,Y,-X,-13,-16

Sample 3 M81-infected cells

Mitosis	Karyotype
1	46,XY
2	46,XY
3	46,XY
4	46,XY
5	45,XY,-21
6	46,XY
7	46,XY
8	43,XY,-2,-21,-21
9	45,XY,-9
10	43,XY,-4,-14,-16
11	46,XY
12	46,XY
13	46,XY
14	45,XY,-22
15	46,XY
16	41,XY,-6,-7,-7,-10,-19
17	44,XY,-12,-15
18	46,XY
19	44,XY,-18,-21
20	44,XY,-7,-16

Sample 4 M81/ΔZR-infected cells

Mitosis	Karyotype
1	46,XY
2	42,XY,-7,-12,-20,-22
3	46,XY
4	46,XY
5	45,XY,-18
6	46,XY
7	46,XY
8	46,XY
9	46,XY
10	45,XY,-8
11	46,XY
12	46,XY
13	46,XY
14	46,XY
15	46,XY
16	46,XY
17	46,XY
18	40,XY,-4,-9,-10,-11,-21,-22
19	45,XY,-21
20	46,XY

Sample 5 M81/ΔZR-infected cells

Mitosis	Karyotype
1	46,XY
2	46,XY,del(4q)
3	46,XY
4	44,XY,-3,tas(14;22)
5	46,XY
6	46,XY
7	44,XY,-3,-14
8	46,XY
9	46,XY
10	46,XY
11	46,XY
12	46,XY
13	47,XY,+Y
14	46,XY
15	47,XY,+Y
16	40,XY,+X,+1,+2,-4,-5,+6,+7,+8,-9,-10, der(11q),-13,-14,-18,-19,-19,-20,-21,-21
17	46,XY,der(14)t(14;14)
18	46,XY
19	46,XY
20	46,XY

Sample 6 M81/ΔZR-infected cells

Mitosis	Karyotype
1	46,XY
2	45,XY,-20
3	46,XY
4	46,XY
5	46,XY
6	46,XY
7	38,XY,-1,-4,-4,-6,-8,-15,-16,-18
8	46,XY
9	37,XY,-2,-3,-3,-5,-6,-7,-8,-14,-19
10	46,XY
11	44,XY,-17,-20
12	46,XY
13	46,XY
14	46,XY
15	46,XY
16	44,XY,-14,-22
17	41,XY,-9,-13,-17,-19,-21
18	46,XY
19	46,XY
20	46,XY

Supplementary Figure 2. Translocations and deletions take place early post-infection but are much less frequent than aneuploidy. (a) Three pairs of cells transformed with M81 or with M81/ΔZR were submitted to a M-FISH analysis at day 6 post-infection. The table shows the frequency of aneuploidy, deletion and translocation, as well as of clonal events in the investigated cells. (b to e) show examples of M-FISH analyses that revealed the presence of translocations and chromosome deletions. (f) This figure shows the detailed results of the M-FISH analyses.

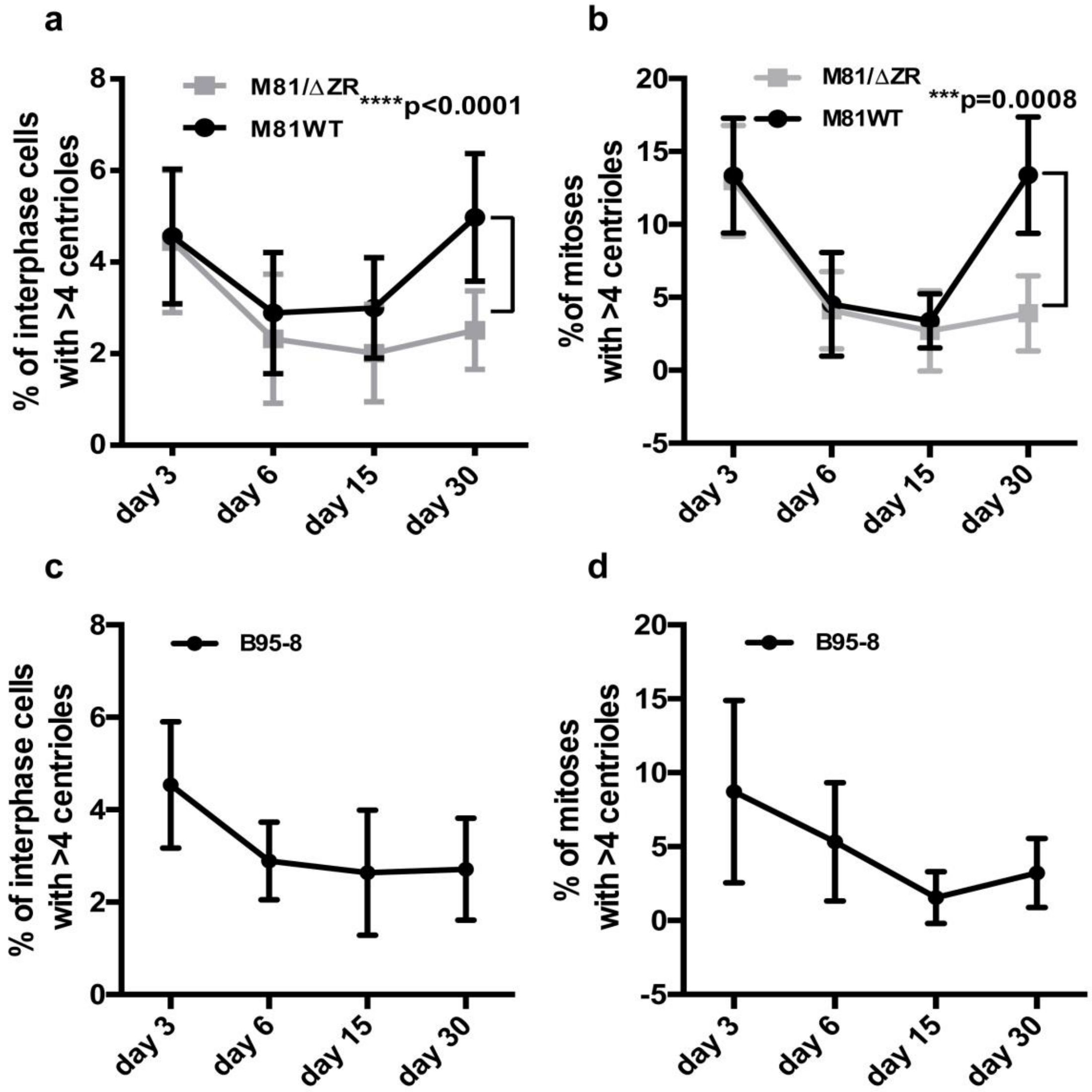

Supplementary Figure 3. Centrosome amplification in EBV-infected cells is visible 3 days after infection, regularly decreases until day 15 and re-increases after onset of lytic replication at day 30. We monitored centrosome amplification in interphase cells and mitotic cells over a period of one month. The graphs show the percentage of interphase cells or the percentage of mitoses with more than 4 centrioles at day 3, 6, 15 and 30 post infection. 8 independent blood samples were infected with M81 and M81/ΔZR (a) and (b), or with B95-8 (c) and (d). The differences between cells infected with M81 or with M81/ΔZR were evaluated by a paired two-tailed t-test ((a) $p<0.0001$, (b) $p=0.0008$). Error bars represent the mean with s.d.

a

Sample	Virus	Aneuploidy rate	Clonality	Deletion rate	Translocation rate
1	M81WT	10/20	no	0/20	0/20
2	M81WT	6/20	no	1/20	1/20
3	M81WT	8/20	yes	1/20	16/20
4	M81WT	7/20	yes	0/20	0/20
	Average	38,75%	2/4	2/4	2/4
5	M81/ΔZR	1/15	no	0/20	0/20
6	M81/ΔZR	0/20	no	0/20	0/20
7	M81/ΔZR	1/20	no	0/20	0/20
8	M81/ΔZR	5/20	no	0/20	0/20
	Average	9%	0/4	0/4	0/4

b

Sample	Virus	Aneuploidy rate	Clonality	Deletion rate	Translocation rate
9	B95-8	4/20	yes	0/20	18/20

c

M-FISH

G-banding

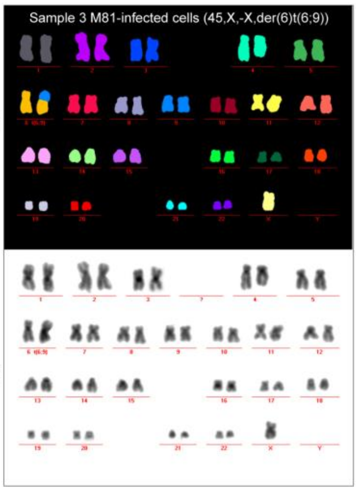

d

M-FISH

G-banding

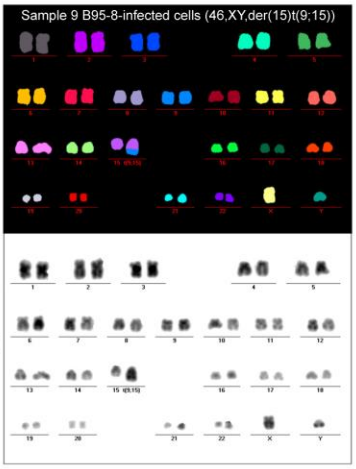

e

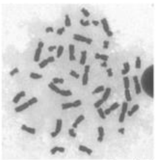

2n-45

f

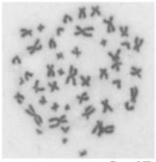

2n-47

g

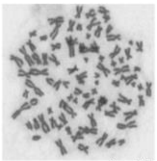

4n-92

h

Sample 1 M81-infected cells		Sample 2 M81-infected cells	
Mitosis	Karyotype	Mitosis	Karyotype
1	45,XX,-18	1	46,XX
2	46,XX	2	46,XX
3	46,XX	3	47,XX,+13
4	45,XX,-1	4	38,XX,-4,-5,-7,-7,-8,-17,-19,-22
5	43,XX,-1,-3,-15	5	41,X,-X,der(1)t(1;2),-3,-7,-14,-19
6	41,XX,-1,-5,-7,-11,-13	6	44,XX,-2,-17
7	44,XX,-2,-4	7	46,XX
8	46,XX	8	46,XX
9	45,XX,-17	9	46,XX
10	46,XX	10	46,XX
11	46,XX	11	46,XX
12	41,X,-X,-1,-2,-7,-14	12	45,XX,del(1q),-6
13	46,XX	13	46,XX
14	46,XX	14	46,XX
15	46,XX	15	46,XX
16	46,XX	16	46,XX
17	42,X,-X,-3,-4,-13	17	46,XX
18	44,XX,-2,-3	18	45,XX,-19
19	46,XX	19	46,XX
20	38,XX,-1,-6,-8,-9,-13,-14,-14,-17	20	46,XX

Sample 3 M81-infected cells		Sample 4 M81-infected cells	
Mitosis	Karyotype	Mitosis	Karyotype
1	45,X,-X,der(6)t(6;9)	1	45,X,-Y
2	45,X,-X,der(6)t(6;9)	2	46,XY
3	45,X,-X,der(6)t(6;9)	3	46,XY
4	44,XX,-4,-19	4	46,XY
5	43,X,-X,der(6)t(6;9),der(15)t(15;16),-21-22	5	46,XY
6	44,X,-X,der(6)t(6;9),-19	6	46,XY
7	45,X,-,X,der(6)t(6;9)	7	46,XY
8	45,X,-,X,der(6)t(6;9)	8	45,X,-Y
9	45,X,-,X,der(6)t(6;9)	9	45,X,-Y
10	90,XX,-,X,-X,der(6)t(6;9)x2	10	45,X,-Y
11	45,X,-,X,der(6)t(6;9)	11	45,X,-Y
12	45,X,-,X,der(6)t(6;9)	12	46,XY
13	45,X,-,X,-4,der(6)t(6;9),-14,-21	13	46,XY
14	47,XX,del(16),+del(16)	14	46,XY
15	40,X,-X,-2,-10,-15,-15,-18	15	46,XY
16	45,X,-,X,der(6)t(6;9)	16	46,XY
17	32,-X,-,X,der(6)t(6;9),-7,-9,-10,-12,-16,-16,-17,-19,-19,-20,-22,-22	17	45,X,-Y
18	42,-X,-X,-15,-21	18	46,XY
19	45,X,-,X,der(6)t(6;9)	19	46,XY
20	39,X,-,X,der(6)t(6;9),-11,-14,-16,-20,-21,-22	20	48,XYYY,+12,-13

Sample 5 M81/ΔZR-infected cells		Sample 6 M81/ΔZR-infected cells	
Mitosis	Karyotype	Mitosis	Karyotype
1	46,XX	1	46,XX
2	46,XX	2	46,XX
3	46,XX	3	46,XX
4	46,XX	4	46,XX
5	43,XX,-1,-3,-19	5	46,XX
6	46,XX	6	46,XX
7	46,XX	7	46,XX
8	46,XX	8	46,XX
9	46,XX	9	46,XX
10	46,XX	10	46,XX
11	46,XX	11	46,XX
12	46,XX	12	46,XX
13	46,XX	13	46,XX
14	46,XX	14	46,XX
15	46,XX	15	46,XX
16		16	46,XX
17		17	46,XX
18		18	46,XX
19		19	46,XX
20		20	46,XX

Sample 7 M81/ΔZR-infected cells		Sample 8 M81/ΔZR-infected cells	
Mitosis	Karyotype	Mitosis	Karyotype
1	46,XY (Double-strand break in 1q)	1	46,XY
2	100,XXYY,+6,+12,+13,+15	2	45,XY-2
3	46,XY	3	45,XY-20
4	46,XY	4	45,XY-20
5	46,XY	5	46,XY
6	41,X,-Y,-17,-19,-22,-22	6	46,XY
7	46,XY	7	46,XY
8	46,XY	8	46,XY
9	46,XY	9	46,XY
10	46,XY	10	45,XY-18
11	46,XY	11	46,XY
12	46,XY	12	43,X,-Y,-18,-21
13	46,XY	13	46,XY
14	46,XY	14	46,XY
15	46,XY	15	46,XY
16	46,XY	16	46,XY
17	46,XY	17	46,XY
18	46,XY	18	46,XY
19	46,XY	19	46,XY
20	46,XY	20	46,XY

Sample 9 B95-8-infected cells	
Mitosis	Karyotype
1	46,XY,der(15)t(9;15)
2	46,XY,der(15)t(9;15)
3	46,XY,der(15)t(9;15)
4	46,XY,der(15)t(9;15)
5	44,X,-Y,-2,der(15)t(9;15)
6	46,XY,der(15)t(9;15)
7	39,XY,-5,-13,-14,der(15)t(9;15),-17,-20,-22,-22
8	46,XY,der(15)t(9;15)
9	46,XY,der(15)t(9;15)
10	46,XY,der(15)t(9;15)
11	46,XY,der(15)t(9;15)
12	46,XY,der(15)t(9;15)
13	46,XY
14	46,XY,der(15)t(9;15)
15	45,XY,der(15)t(9;15),-22
16	38,X,-Y,-6,-9,-10,-11,-14,-der(15)t(9;15),-18
17	46,XY,der(15)t(9;15)
18	46,XY,der(15)t(9;15)
19	46,XY,der(15)t(9;15)

Supplementary Figure 4. Cells infected with wild type EBV exhibit a higher aneuploidy rate and a higher frequency of clonal abnormalities than cells infected with a replication-defective virus. (a) Four pairs of cells transformed with

M81 or with M81/ΔZR were submitted to a M-FISH analysis between 4 and 6 weeks post-infection. The table shows the frequency of aneuploidy, deletion and translocation, as well as of clonal events in the investigated cells. (b) shows the same analysis for cells infected with B95-8 at day 60 post-infection. (c) and (d) show examples of M-FISH analyses that revealed the presence of translocations. (e to g) The figures show examples of Giemsa-stained mitotic plates from cells infected with wild type M81. (h) This figure shows the detailed results of the M-FISH analyses.

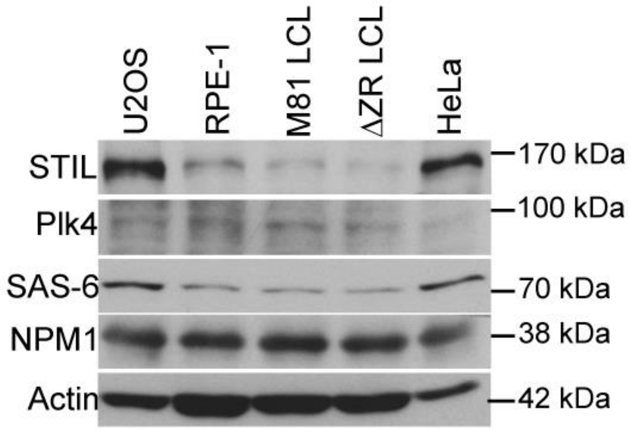

Supplementary Figure 5. Comparative expression levels of proteins involved in centrosome duplication in cells of different lineages. We performed immunostains on a panel of cells including EBV-infected cells and three different cell lines (U2OS, RPE-1, HeLa), with antibodies specific to Plk4, SAS-6 and STIL. Immunoblots with antibodies specific for actin and NPM1 served as loading controls. Please also see uncropped full blots in Supplementary Fig. 12.

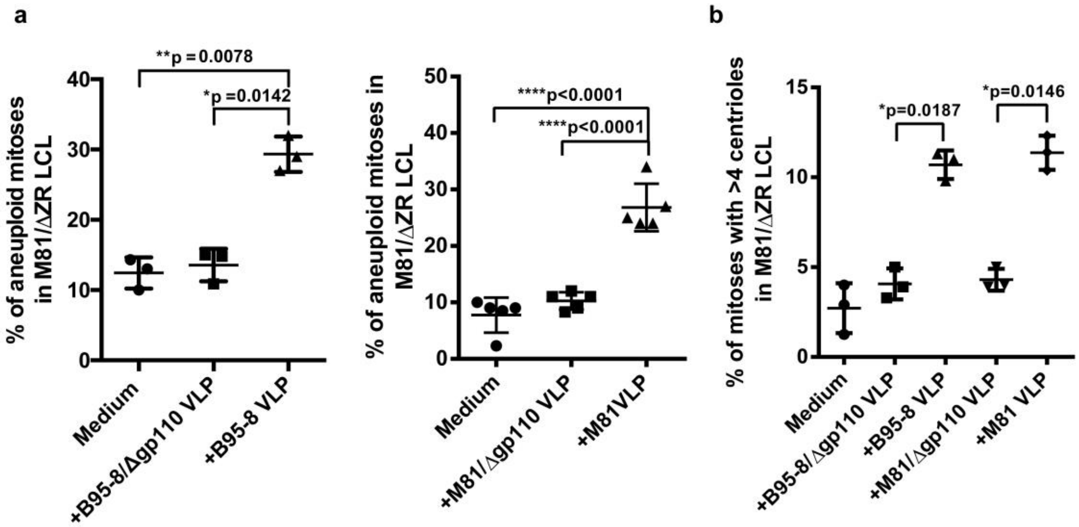

Supplementary Figure 6. VLPs from B95-8 or M81 display identical properties. (a) The graphs show the percentage of aneuploidy in cells transformed by M81/ΔZR exposed to culture medium, to wild type VLPs from B95-8 or M81, or to VLPs from these two viruses devoid of the gp110 protein. We treated 3 independent LCLs with B95-8 VLPs and 5 LCLs with M81 VLPs. (b) The dot plots display the rate of centrosome amplification in 3 independent LCLs established with M81/ΔZR and treated with VLPs under the conditions described in (a). The results of paired two-tailed t-tests ((a) p=0.078 and 0.0142, (b) both p<0.0001, (c) p=0.0187 and 0.0146). Error bars represent the mean with s.d.

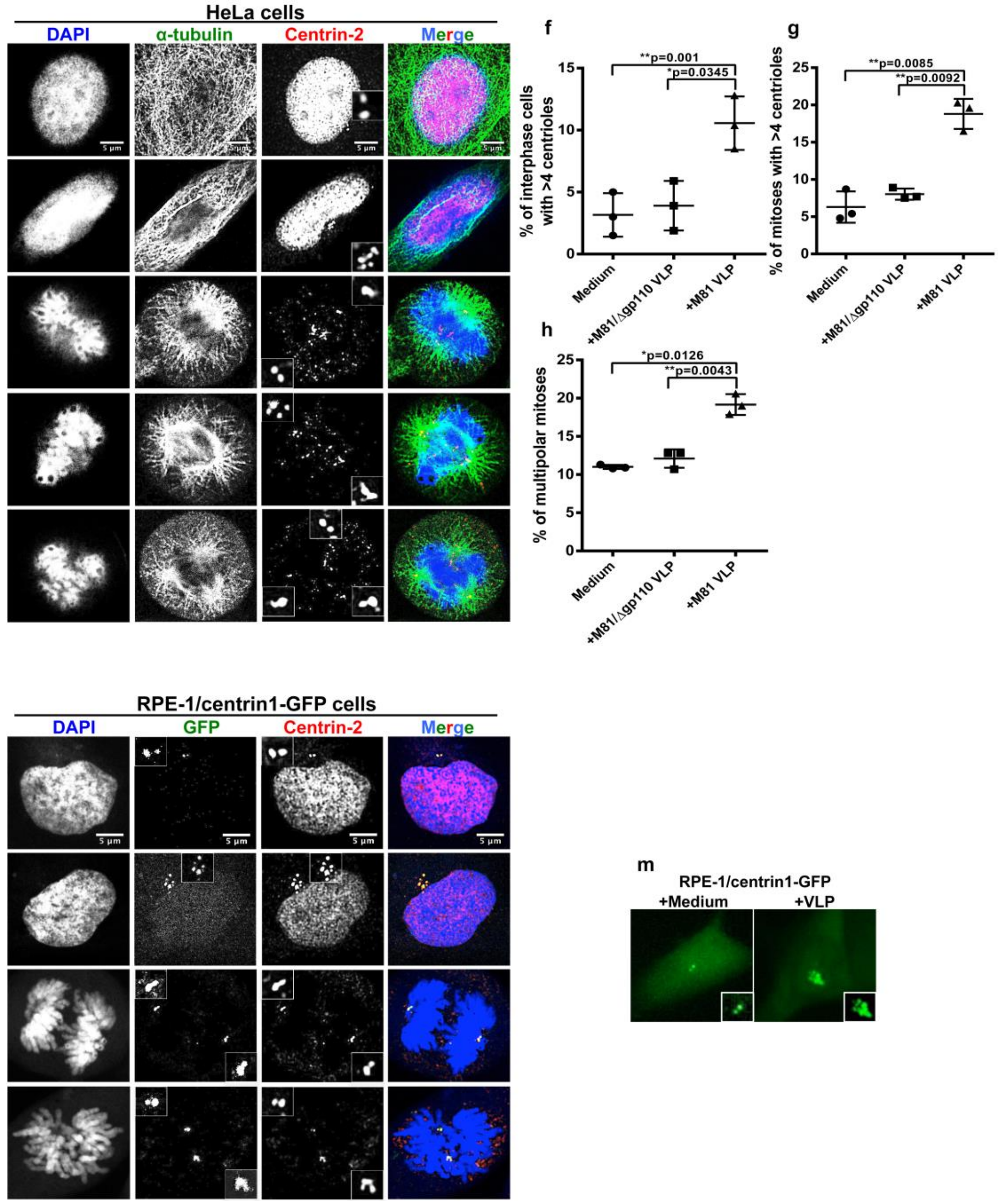
HeLa cells
DAPI
α-tubulin
Centrin-2
Merge
a
b
c
d
e
5 µm
f
% of interphase cells with >4 centrioles
**p=0.001
*p=0.0345
Medium
+M81/Δgp110 VLP
+M81 VLP
g
% of mitoses with >4 centrioles
**p=0.0085
**p=0.0092
h
% of multipolar mitoses
*p=0.0126
**p=0.0043
RPE-1/centrin1-GFP cells
DAPI
GFP
Centrin-2
Merge
i
j
k
l
m
RPE-1/centrin1-GFP
+Medium
+VLP

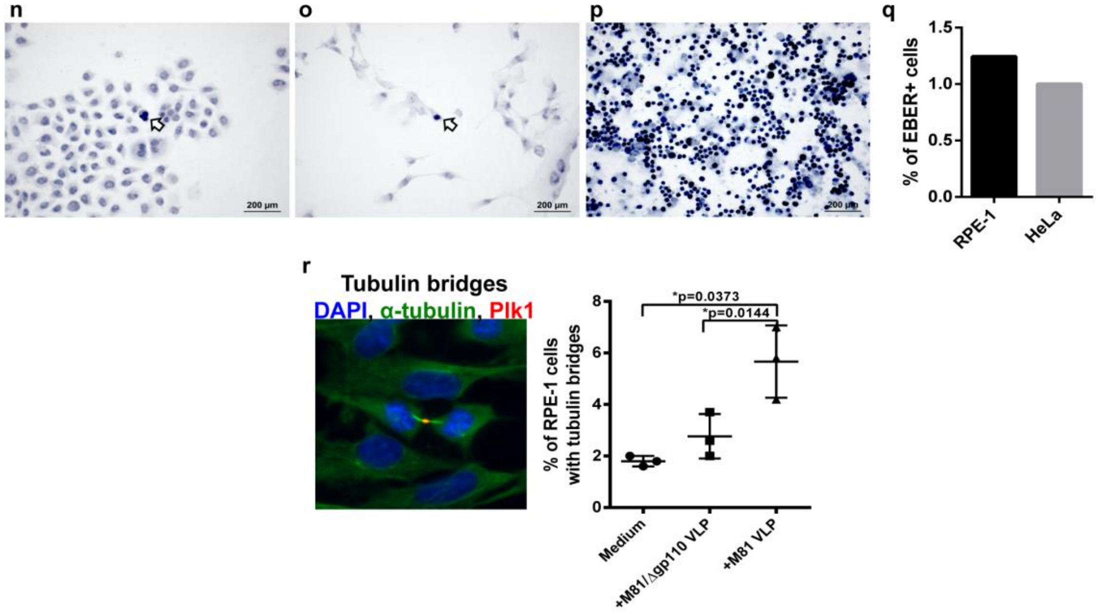

Supplementary Figure 7. Exposure of RPE-1 cells or HeLa cells to EBV VLPs induces centriole amplification. (a to h) HeLa cells were treated with M81 EBV VLPs or M81/Δgp110 VLPs and stained with antibodies specific to alpha-tubulin and centrin-2. The pictures show examples of interphase HeLa cells with 2 centrioles (a) or more than 4 centrioles (b). We also show mitoses with 4 (c) or more than 4 centrioles (d). (e) shows a cell undergoing a tripolar mitosis. Scale bar, 5 μm. (f to h) the graphs show the frequency of interphase cells with more than 4 centrioles (f), of bipolar mitoses organized around more than 4 centrioles (g), and of multipolar mitoses (h). The results of three experiments were analysed with a paired two-tailed t-test ((f) p=0.001 and 0.0345, (g) p=0.0085 and 0.0092, (h) p=0.0126 and 0.0043). (i to m) We treated RPE-1 cells that carry a centrin1-GFP fusion protein with M81 VLPs. Cells were then stained with an antibody specific to centrin-2 three days after infection to confirm co-localization with the GFP signals. (i) shows one interphase cell with 2 centrioles, (j) one interphase cell with multiple centrioles, (k) one mitosis with 4 centrioles, (l) one mitosis with more than 4 centrioles, (m) shows pictures of live RPE-1 cells that carry a centrin1-GFP fusion protein and were treated with medium or with VLPs for three days. (n-q) We infected HeLa cells (n) or REP-1 cells (o) with wild type M81 viruses and evaluated the infection rate using an *in situ* hybridization directed against the EBV non-coding RNA EBER. LCL cells transformed with M81 virus served as a positive control (p). (q) The infection rates are shown in a bar graph. (r) Here we stained the cells for alpha-tubulin and Plk1 three days post-infection to visualize cells undergoing cytokinesis. The picture shows an example of a RPE-1 cell undergoing cytokinesis and the graph shows the frequency of cells with tubulin bridges after treatment with different types of EBV VLPs. The results of 3 independent experiments were analysed with a paired two-tailed t-test ((r) p=0.0373 and 0.0144). Scale bar in a-e and i-l, 5 μm, in n-p, 200 μm. Error bars represent the mean with s.d.

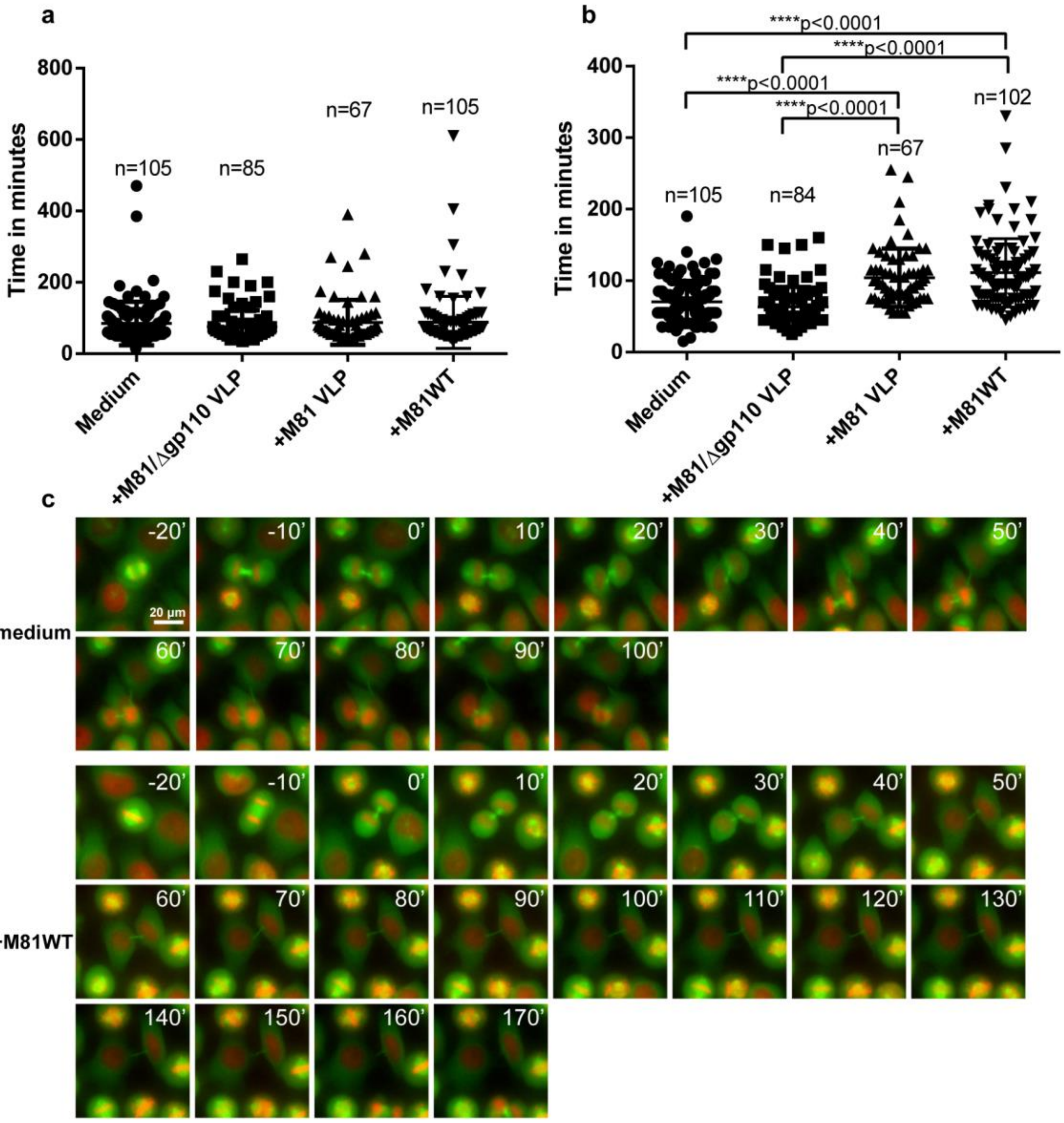

Supplementary Figure 8. Live cell imaging of HeLa Kyoto mEGFP-α-tubulin/H2B-mCherry cells treated with wild type (WT) M81, wild type virus-like particles (VLP) or virus-like particles that lack gp110. The figure shows the average time for completion of mitosis (a) and the time for completion of cytokinesis (b). The data in (a) and (b) were log-transformed and subjected to an ANOVA test whose results are given if the test showed significant differences. We show Bonferroni-adjusted pairwise comparisons ((b) $p<0.0001$, n: number of analysed mitoses). (c) shows still images of the live cell imaging with sequential time points (minute) whereas 0 min defines the end of telophase after treatment with medium or wild type M81. In each case we show a cell undergoing cytokinesis. Scale bar, 20 µm. Error bars represent the mean with s.d. Please also watch the Supplementary Movies 1 and 2.

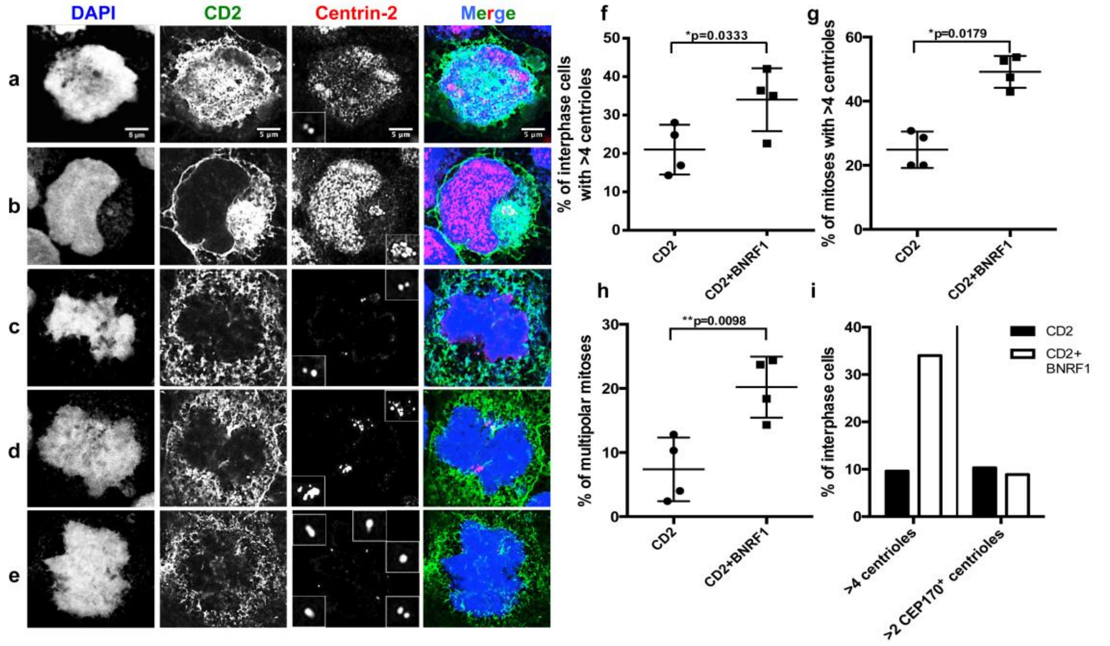

Supplementary Figure 9. Transfection of BNLF1 leads to centrosome amplification in 293 cells. The figure shows 293 cells cotransfected with BNRF1 and CD2 stained with antibodies specific to CD2 and centrin-2. Scale bar, 5 µm. (a) and (c) show interphase cells and mitotic cells with 2 and 4 centrioles. We also show examples of centriole amplification in interphase (b) and mitotic cells (d), as well as a multipolar mitosis (e). (f-h) The dot plots show the frequency of interphase (f) or mitotic cells (g) with centriole amplification and the frequency of multipolarity (h) in cells transfected with CD2 only or CD2 together with BNRF1. We applied a paired two-tailed t-test to the results of three independent experiments ((f) p=0.0333, (g) p=0.0179, (h) p=0.0098). (i) The graph of bars shows the percentage of cells with more than 4 centrioles or more than 2 CEP170-positive centrioles in 293 cells transfected with CD2 or co-transfected with CD2 and BNRF1. Error bars represent the mean with s.d.

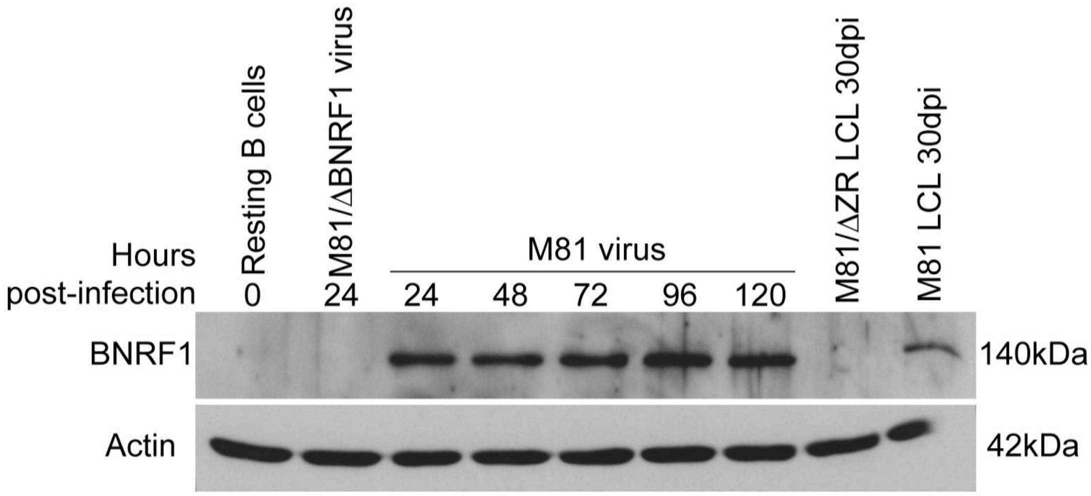

Supplementary Figure 10. BNRF1 expression after infection of B cells with wild type EBV. Primary B cells were exposed to wild type M81 virus. BNRF1 protein level in these cells was assessed by immunoblot with a BNRF1-specific antibody from day 1 to day 5 post-infection. We also included a pair of one-month-old LCLs established with wild type M81 or with the M81/ΔZR virus. Non-infected B cells and B cells transformed by the ΔBNRF1 virus provided negative controls. Please also see Supplementary Fig. 12.

Figure. 6e

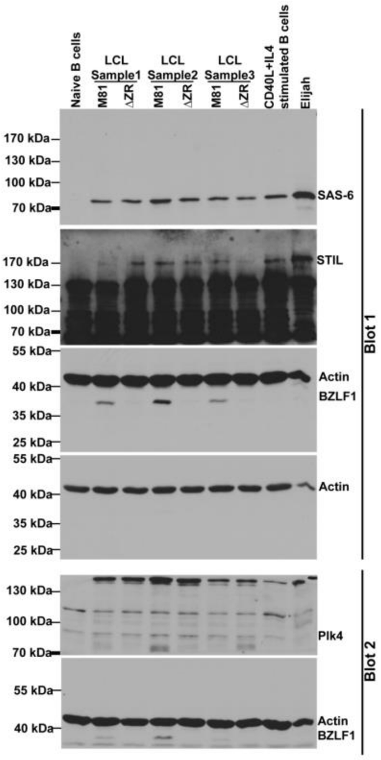

Figure. 9

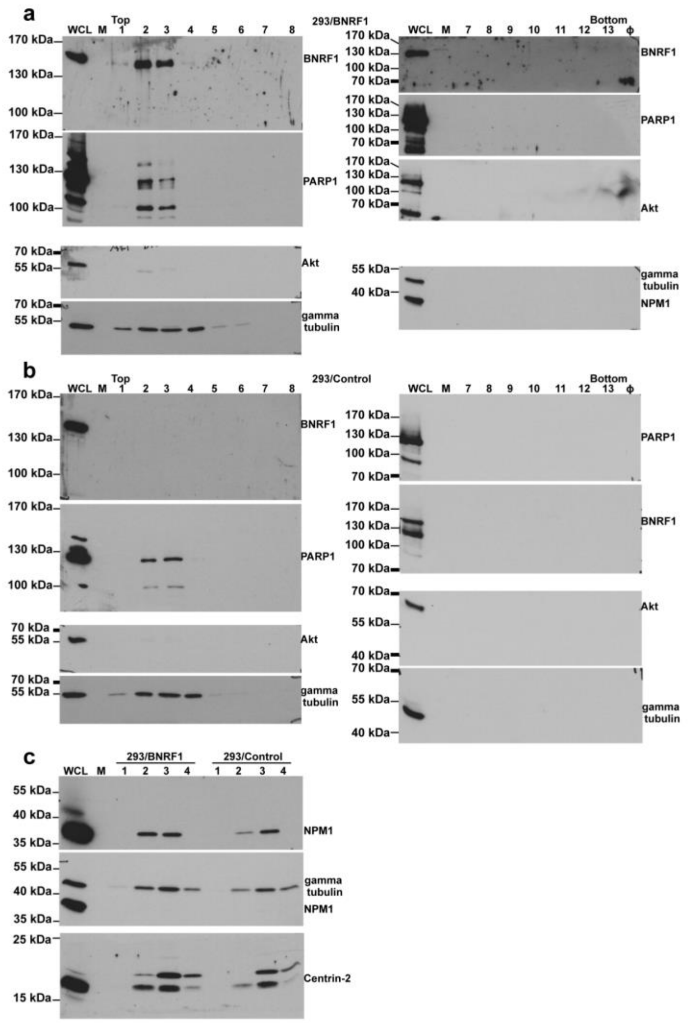

Supplementary Figure 11. Original images of Western blots shown in main figures. We show here the full blots that were cropped in the previous figures. When multiple antibodies were tested on the same membrane, we show the different blots in chronological order from top to bottom.

Supplementary Figure S5

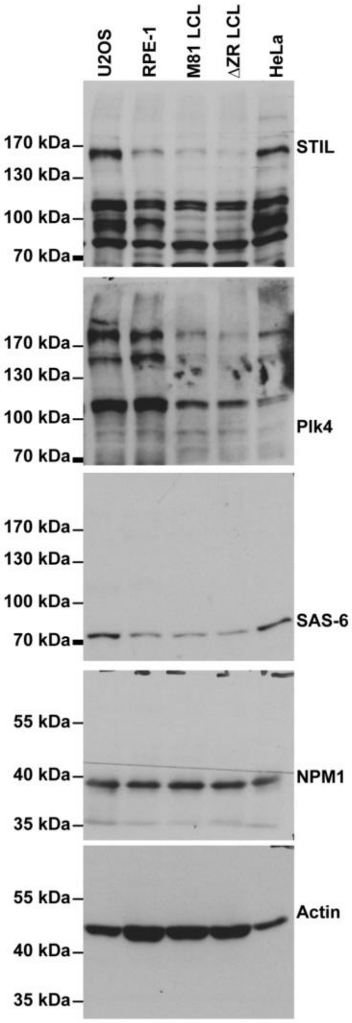

Supplementary Figure S10

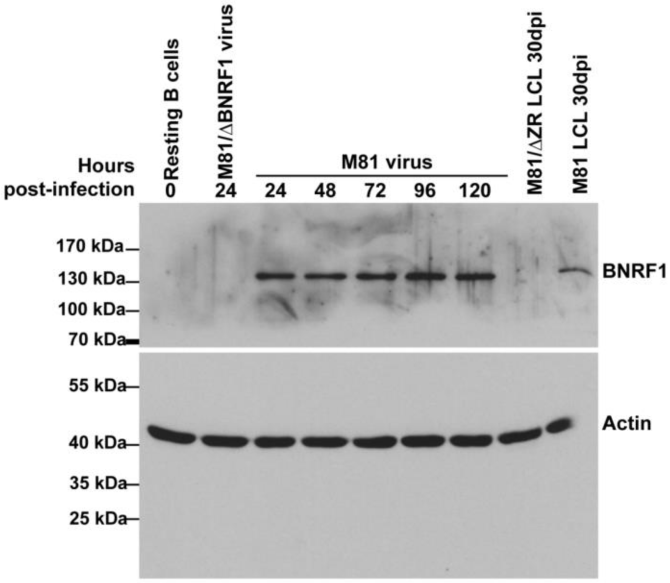

Supplementary Figure 12. Original images of Western blots shown in Supplementary figures. We show here the full blots that were cropped in the previous supplementary figures. When multiple antibodies were tested on the same membrane, we show the different blot in chronological order from top to bottom.
